# An Epidemic

**Published:** 1903-11

**Authors:** 


### An Epidemic.

The Copenhagen papers publish this piquant little story:

A prominent business man appeared at the office of his family physician and communicated to him with great concern that his son, the joy and hope of the family, to all appearances was suffering from diphtheria.

The doctor shrugged his shoulders in a sympathetic way: "Very sorry to hear it. No mother's soul is safe when that sneaking disease comes around."

"But," continued the man, "the dear young lad has confessed that he caught the disease from the house-maid, whom he had kissed."

"Well, what in the world shall one say to that? Young people are very thoughtless," remarked the doctor, discreetly.

"But, don't you see, doctor—how—to be plain—between you and me—I have also kissed the girl (the horrid thing); perhaps I, too, will be down with the disease."

"Yes, by thunder, that is the next thing to expect——"

"And I kiss my dear wife every morning and evening, so we risk having her——"

"Gracious goodness," exclaimed the doctor, bringing his fist down with emphasis, "then I, too, will have it!"

Tableau.

